# New Directions in Understanding Atopic March Starting from Atopic Dermatitis

**DOI:** 10.3390/children9040450

**Published:** 2022-03-23

**Authors:** Nunzia Maiello, Pasquale Comberiati, Arianna Giannetti, Giampaolo Ricci, Rossella Carello, Elena Galli

**Affiliations:** 1 Department of Woman, Child and General and Specialized Surgery, University of Campania “Luigi Vanvitelli”, 81100 Naples, Italy; 2 Department of Clinical and Experimental Medicine, Section of Pediatrics, University of Pisa, 56126 Pisa, Italy; pasquale.comberiati@gmail.com; 3 Department of Clinical Immunology and Allergology, I.M. Sechenov First Moscow State Medical University, 119991 Moscow, Russia; 4 Pediatric Unit, IRCCS Azienda Ospedaliero-Universitaria di Bologna, 40138 Bologna, Italy; ariannagiannetti25@gmail.com; 5 Department of Medical and Surgical Sciences (DIMEC), University of Bologna, 40138 Bologna, Italy; giampaolo.ricci@unibo.it; 6 Pediatric Allergic Unit, S.Pietro Hospital FbF Roma, 00189 Rome, Italy; carellorossella@gmail.com (R.C.); galli.elena@fbfrm.it (E.G.)

**Keywords:** atopic march, atopic dermatitis, food allergy, asthma, allergic rhinitis, eosinophilic esophagitis

## Abstract

Recent evidence showed that the postulated linear progression of the atopic march, from atopic dermatitis to food and respiratory allergies, does not capture the heterogeneity of allergic phenotypes, which are influenced by complex interactions between environmental, genetic, and psychosocial factors. Indeed, multiple atopic trajectories are possible in addition to the classic atopic march. Nevertheless, atopic dermatitis is often the first manifestation of an atopic march. Improved understanding of atopic dermatitis pathogenesis is warranted as this could represent a turning point in the prevention of atopic march. In this review, we outline the recent findings on the pathogenetic mechanisms leading to atopic dermatitis that could be targeted by intervention strategies for the prevention of atopic march.

## 1. Introduction

**Figure 1:**
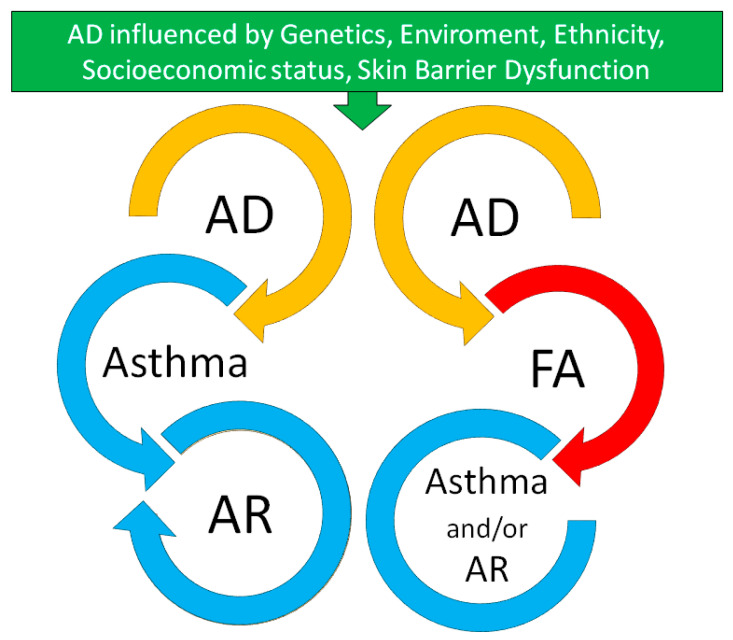
Allergic march trajectories starting from atopic dermatitis. AD, atopic dermatitis; AR, allergic rhinitis; FA, food allergy.

The model of atopic march was originally proposed to describe the natural history of atopic manifestations. According to this model, atopy starts in infancy with atopic dermatitis (AD) and progress to asthma and/or allergic rhinitis (AR) in childhood and adolescence, with the potential development of food allergy (FA) before respiratory allergies in a subgroup of individuals (Figure 1) [1].

AD often appears early in life, and thus it has been considered as the origin of the atopic march by most prospective longitudinal cohort studies looking at trajectories of allergic diseases [2]. Prospective longitudinal cohort studies have shown that about 50% of children with AD does not progress into the atopic march, even in its broadest definition [3]. Indeed, recent evidence from birth cohort studies, that applied the Bayesian machine learning framework, shows that the postulated linear progression of the atopic march does not capture the heterogeneity of allergic phenotypes, which are influenced by a complex interaction between environmental, genetic, and psychosocial factors (Figure 1) [4].

Nevertheless, AD is often the first manifestation of an atopic march. Improved understanding of AD is warranted as this could represent a turning point in the prevention of allergic diseases. In this review, we outline the recent findings on the pathogenetic mechanisms leading to AD that could be targeted by intervention strategies for the prevention of atopic march.

## 2. The Atopic March: Myth or Reality?

Despite the existence of epidemiological and experimental data in support of the atopic march, some researchers have highlighted the risk of an overestimation of the classical march [5].

First, disease identification in existing epidemiological investigations is at risk of bias, because data on the diagnosis of AD, asthma, and AR are commonly self-reported reported by caregivers and not necessarily physician-confirmed atopic condition [6,7,8].

Another critical issue is the inability to address the heterogeneity of atopic diseases. According to Martinez et al. [6] individuals with AD were at higher risk of developing transient early asthma and persistent asthma, but non-allergic late-onset asthma. This could indicate that the association between AD and asthma may be limited to specific allergic asthma subpopulations. A recent study used Bayesian machine learning and latent class analysis to examine data from two birth cohorts and highlighted the heterogeneity in the trajectories of allergic diseases development [4]. These authors identified eight latent classes: no disease (51.3%), eczema only (15.3%) and rhinitis only (9.6%), transient wheezing (7.7%), persistent wheezing with late-onset rhinitis (5.7%), atopic march (3.1%), persistent eczema and wheezing (2.7%), persistent eczema with late-onset rhinitis (4.7%). The persistence of AD (after several consecutive visits) was essentially related to asthma or rhinitis. Highly concordant sensitization patterns have been associated with different profiles of rhinitis, eczema, and wheezing. In each case, it was found that eczema, wheezing and rhinitis are all common pathologies, which therefore coexist, but above all as separated entities, each related to an extent higher or lower atopic sensitization. Although some children follow trajectories similar to the atopic march, this study shows that the classical march is detectable in about one in 20 children with atopic symptoms [9].

## 3. Atopic March Risk Factors

### 3.1. Age of Onset and Severity of Atopic Dermatitis

The age of AD onset and its severity influence the relationship between AD and allergic airway disease. Multimorbidity and more persistent disease are more commonly experienced by patients with more severe AD. The severity of AD is the strongest risk factor for early-onset AD [10]

High-risk children in the MACS study with persistent early-onset AD had a 3-fold greater risk of developing asthma and AR in late childhood compared to infants with late-onset AD starting after 2 years of age [11].

In another Swedish study, more than 60% of infants with severe AD before age 3 developed asthma by age 7, compared with only 20% of those with mild AD [12]. Increased asthma severity and greater persistence into adulthood have also been associated with the presence of AD [6,13]. Similar results were reported in the PASTURE cohort [14], the PEER study [15], and the BAMSE cohort [16].

In an Italian cohort that followed 252 children (ages 6–36 months) for an average of 16.9 years, the severity of AD was significantly related to the development of asthma [17].

Roduit et al. [14] compared children of women who lived on farms vs. those who did not live on farms in the Protection Against Allergy: Study in Rural Environments (PASTURE). These researchers identified 4 phenotypes of AD in childhood: 2 early phenotypes with onset before 2 years (early transient (*n* = 96; 9.2%) and early persistent (*n* = 67; 6.5%)), one phenotype with onset at age 2 years or older (*n* = 50; 4.8%), and the never/infrequent phenotype (*n* = 825; 79.5%), recognized as children without AD. Both early phenotypes were strongly related to FA. The early persistent phenotype had also a significantly increased risk of developing asthma (adjusted odds ratio, 2.87; 95% CI, 1.31–6.31). The late phenotype was only positively associated with the risk of AR.

Paternoster et al. [18] examined AD trajectories from birth to adolescence in 9894 children from the Child Avon Longitudinal Study of Parents and Children (ALSPAC) cohort study and 3652 children from the Prevention and Incidence of Asthma and Mite Allergy (PIAMA) cohort study. Six latent classes were identified: (1) unaffected/transiently affected, (2) early-onset/early resolution (most common AD class), (3) early onset/late resolution, (4) early-onset/persistent, (5) late-onset/resolution, and (6) medium onset/resolution. The more persistent classes (persistent and late resolution) were most strongly related with filaggrin (FLG)-null mutations and showed the greatest risk of coexistence of asthma, high IgE levels, and parental history of atopy. The probability of asthma was highest for the early-onset/persistent class at age 7 (OR, 5.50; 29% vs. 8% in the normal/transient class) and age 13 (OR, 7.9; 31% vs. 7%) and weaker for the early-onset/early resolution class (OR, 1.56 at 7 years and 1.79 at 13 years).

### 3.2. Allergic Polysensitization

A recent systematic review reported a strong and dose-dependent association between AD and IgE-mediated FA [19].

Children with moderate to severe AD are more likely to have FA and associated respiratory allergies [20,21].

The Mechanisms of the Development of Allergy (MeDALL) study reported a rare but severe phenotype in which individuals who were polysensitized and with multimorbidity have a very high frequency, higher than other phenotypes, of more severe and persistent AD symptoms and total and specific IgE levels [22].

In another BAMSE cohort, 62% of aeroallergen-sensitized children aged 4 to 16 years had concomitant eczema, rhinitis, or asthma [23].

The PARIS (Pollution and Asthma Risk: An Infant Study) birth cohort recently reported that allergic sensitization in infancy can predict allergic multimorbidity in childhood, and in the case of early polysensitization, multimorbidity is more frequent as soon as infancy [24].

The MACS (Melbourne Atopy Cohort Study, high risk) and LISAplus (Influences of Lifestyle-Related Factors on the Immune System and the Development of Allergies in Childhood plus Air Pollution and Genetics) population cohort studies reported that sensitization to food allergens in the first 24 months of life is associated with a higher risk of asthma and AR at the age 10 years [25]. A meta-analysis of 13 cohorts showed that an increased risk of wheezing/asthma and AR between 4 and 8 years was associated with early food sensitization [26]. Hill et al. [27] noted that children with FA, particularly milk, egg, and peanut allergies, were at increased risk of developing rhinitis and asthma in late childhood. The Canadian Healthy Infant Longitudinal Development birth cohort study showed that there is an increased risk of physician-diagnosed asthma at 3 years only if the AD is associated with sensitization to inhalants, foods, or both at 1 year of age [28].

The birth cohort study WHEALS (Wayne County Health, Environment, Allergy, and Asthma Longitudinal Study) looked at the relationship between early allergic sensitization (specific IgE to 10 inhalant and food allergens measured at age 2) and the risk of pediatric asthma and allergic diseases at 10 years of age. Four latent classes were identified based on sensitization profiles at the age of 2: (a) highly sensitized, (b) milk/egg dominant, (c) peanuts and inhalants, and (d) low-to-zero sensitization. At age 10, an allergist screened children for ongoing AD and asthma through interviews and a physical exam. Methacholine challenge test and spirometry were also performed. Compared to the group “low-to-zero sensitization”, infants sensitized to ≥4 food and inhalant allergens at the age of 2 had the highest risk of current asthma (hazard ratio = 4.42) *p* < 0.001) and bronchial hyper reactivity (HR 1.77; *p* < 0.001). The risk of current AD was independent of the sensitization pattern but remained raised for children with any sensitization. No differences were found in spirometry parameters [29].

### 3.3. Family History for Atopy

The probability of subsequent allergic manifestations of the upper or lower airways is increased by a history of childhood AD along with a family history of parental atopy, hence suggesting a genetic influence in the atopic march [30].

In addition, the persistence and severity of atopic conditions such as asthma is strongly predicted by a family history of atopy [11,31,32].

According to the PARIS study, parental history of asthma and/or AR and/or eczema was related to a severe atopic phenotype [33]. In the PASTURE study [14], parental allergy status was strongly related to the early persistent phenotype, and participants with both parents having an allergy history had nearly six times greater risk of following early persistent AD than those with no family history of atopy. However, only a minor proportion (<5%) had AD, asthma, and rhinitis at any age, among those with or without an allergic parent, suggesting that progression from AD to asthma to AR is not a common event [34]. However, it should be noted that these analyses were made on community-based populations that take into account milder forms of AD and asthma.

### 3.4. Genetic Factors

Allergic diseases are strongly influenced by genetics [35]. Many of the genes that have been related to the development of AD are situated on chromosome 1q21, in a position referred as to the epidermal differentiation complex (EDC) [36]. These genes code for proteins important for epithelial keratinocytes maturation and skin barrier function. The strongest genetic risk factor known for AD is the loss-of-function (LOF) mutation in the filaggrin (FLG) gene, which is located in the EDC [37]. The early-onset, persistent AD phenotype is strongly related to FLG null mutations which play an important role in epidermal barrier function, water retention, and acidification. Children with FLG variants who develop asthma are likely to have AD as their first atopic disease. Individuals with AD and FLG mutations are those who are most likely to follow trajectories of severe, persistent, and multimorbid disease [38]. This relationship supports the existence of an endotype-based atopic march and the role of barrier dysfunction in AD and allergic diseases development. FLG mutation-related AD shows distinctive phenotypic features, which include: early-onset AD, increased AD severity, persistent disease, the predilection of cheek and hand lesions, palmar hyperlinearity, increased risk of herpetic eczema and *Staphylococcus (S.) aureus*-mediated skin infections, increased risk of allergic sensitization and asthma (including treatment-resistant asthma), and FA occurrence [38,39].

FLG2 and SPRR3 are 2 other genes located within the EDC that have been implicated in AD. Both genes encode proteins that are important for the structure of the epidermal barrier [40]. Other non-EDC-localized genes identified in AD patients include SPINK5, CLDN1, and TMEM [41,42], which encode proteins that contribute to epidermal homeostasis and barrier function.

A GWAS study showed that AR, asthma, and AD may coexist because they share genetic risk loci that result in dysregulation of immune-related genes [43]. Another GWAS study also found genetic loci that overlapped between asthma and AD [44]. A multi-stage GWAS study in children with childhood AD and asthma found new genetic loci (rs9357733 positioned in EFHC1 on chromosome 6p12.3 and rs993226 between TMTC2 and SLC6A15 on chromosome 12q21.3) that were specific for AD-asthma march phenotype [45]. The Greater Cincinnati Pediatric Clinic Repository (GCPCR) found a relationship between a dependent genetic variant family member Kinesin 3A (KIF3A) with AD-asthma comorbidity in a population cohort [46]. KIFA3A is a component of primary and motile cilia. Deletion of KIF3A in epidermal cells results in disrupted keratinocyte differentiation in animal models, suggesting a role for KIF3A in skin barrier function in addition, deletion of KIF3A in mouse airway epithelial cells causes increased airway hyperresponsiveness and inflammation [46,47,48].

In a recent human study, the KIF3A rs12186803 risk allele differentially interacts with the sensitization pattern to modify asthma risk, resulting in a high risk of asthma even without clinical eczema [49]. These findings suggest that a set of immunologically important genes are shared in the predisposition to multiple allergic manifestations, with some genetic variants showing a different impact on allergic outcomes.

In summary, although important progress has been achieved in identifying allergic risk loci, studies evaluating genotypic associations with specific allergic trajectories of interest are needed. Gene-environmental interactions are also important because environmental inputs can influence gene transcription through hereditary epigenetic regulation that does not require alterations in the gene sequences [50].

## 4. Pathogenic Mechanisms Underlying Atopic Dermatitis and Possibly Atopic March

### 4.1. Outside-In vs. Inside-Out Hypotheses

The pathogenesis of AD combines a complex interplay of genetic background, environmental influences, immune dysregulation, impaired epidermal barrier, and reduced microbiota diversity. However, the relative contribution of each of these components is yet to be determined. With regards to immune abnormalities, AD is currently considered a biphasic T-cell mediated disease. A Th2 signal predominates in the acute phase whereas a Th2 to Th1 switch promotes disease chronicity [51,52]. Recent studies have proposed a significant role for IL-22 producing T cells and to a fewer extent IL-17 producing Th17 cells, in the initiation and maintenance of AD [51]. Although it is generally recognized that immune dysregulation contributes to AD pathogenesis and disease perpetuation (*inside-out hypothesis*), emerging evidence suggests that a disrupted skin barrier in AD (involving both lesioned and unaffected skin) plays a key role in disease initiation by promoting foreign antigen penetration (e.g., dust mite and food allergens) and subsequent activation of innate and type 2 immune responses (*outside-in hypothesis*) [53].

The skin is the main defense barrier against external stimuli, such as environmental pollutants, ultraviolet light, and pathogens. As a component of the innate immune system, the skin has various defensive functions, enclosing chemical, microbial, physical, and immune barriers [54]. In an organ such as the skin, local breakdown of barrier integrity can cause a systemic breakdown of immune tolerance even in distant organs such as the lungs and intestines.

Dysfunction of the skin barrier can occur even without eczema, as recently reported in some infants who showed subclinical eczema endotype [55]. This suggests the possibility that children and infants without a diagnosis of eczema may have subclinical eczema with normal-looking skin, but with skin dysbiosis, low FLG expression, and increased expression of skin alarmins. These alterations could favor the development of allergic disease without overt eczema [56]. Individuals with AD exhibit epidermal dysfunction with increased transepidermal water loss (TEWL) even in clinically healthy skin sites, which increases the risk of allergen sensitization [55]. Both lesional and non-lesional skin of children with AD exhibits reduced long-chain sphingolipids and aberrant skin metabolism mainly mediated by type 2 inflammation cytokines [56]. Children with skin barrier defects are at a higher risk of asthma than healthy children, even in the absence of AD, indicating even when the allergic skin inflammation is absent the skin may serve as a site for allergen sensitization [57]. Another study showed that uninjured skin of children who suffered from AD and FA has substantially lower filaggrin expression and a lower proportion of waxy lipid molecules such as sphingosine ceramide compared to children with AD only and non-atopic controls. Furthermore, AD/FA + children have greater TEWL than controls. An increased expression of type 2 inflammation related-genes was identified in the skin transcriptome of patients AD/FA +, together with a high expression of the skin proteins keratin 5, 14, and 16, indicating hyperproliferative keratinocytes [58].

Recent pilot studies suggested that intensive emollient use in early life could reduce the risk of developing AD later in life, especially in high-risk infants [59]. However, a very recent systematic review of randomized controlled studies concluded that the use of emollients in healthy infants during the first year of life is probably not an effective strategy for preventing AD and probably increasing the risk of skin infection [59].

### 4.2. The Exposome Hypothesis

This hypothesis supposes that environmental exposure to toxic substances related to the modern lifestyle can affect the epithelial barrier of both the skin, the airways, and the gastrointestinal tract. These toxic substances enclose cleaning products, detergents, microplastics, nanoparticles, ozone, and particulate matter in raised concentrations, cigarette smoke, and food additives (enzymes and emulsifiers) [60]. The role of microplastics, currently omnipresent in the environment, on human health is not fully known. They are present in the air, water, and food. They have been found, for example, in human lungs and feces. The ingestion of microplastic is implicated in intestinal dysbiosis and alterations of the gut microbiome are known to promote inflammatory responses [61]. In addition, histamine levels are shown to increase after high exposure to polypropylene microplastics (~20 and 25–200 μm) [62]. Chronic low-dose exposure to microplastics has the potential to cause impaired intestinal barrier function and epithelial cell injury [63]. In an epithelial cell study of the human lung, it has been reported that exposure to microplastics such as inhaled polystyrene leads to inflammatory and oxidative damage along with the breakdown of intercellular junction proteins in the lung, which would lead to lung barrier dysfunction [64].

Exposure to these harmful agents may lead to the development of inefficient epithelial barriers, translocation of bacteria to the inter-and sub-epithelial areas, microbial dysbiosis, and micro-inflammation of the tissues [60]. According to this hypothesis, the barrier damage caused by environmental changes is not only responsible for the development of autoimmune and allergic diseases, but also for a great range of diseases in which immune responses to translocated bacteria have systemic effects [60]. The dysregulated epithelial barrier activity increases the potential for microbe and allergen penetration and subsequent sensitization. Interactions between microbial, viral, genetic, environmental, and immune factors contribute to epithelial destruction, aberrant Th2 immune responses, and allergic diseases [61]. Deficiencies in the epithelial barrier often drive to a state of constant inflammation, making tissue repair difficult. Due to the inflammatory environment, deficient barrier integrity lowers the sensitization threshold to harmless substances and likely triggers allergic sensitization in distal organs [60].

Environmental pollutants such as carbon monoxide, nitrogen dioxide (NO_2_), and particulate matter 2.5 (PM_2.5_) from fires and pollution has been found to favor acute asthma exacerbations [65,66].

The mechanisms linking asthma exacerbation to air pollutants include induction of both eosinophilic and neutrophilic inflammation driven by stimulation of the epithelium of the airways and increased production of proinflammatory cytokines, oxidative stress, and alterations in DNA methylation [67,68].

Current data demonstrate that the epithelial barriers, such as skin, airways, and intestinal mucosa, can be damaged by pollutants such as cigarette smoke, particulate matter, diesel exhaust, ozone, nanoparticles, microplastics, detergents, and chemicals in household substances. The local inflammation begins, and the tissue can become more vulnerable to the inflammatory and tissue-destructive effects of pollution after the barrier opens. Most of these pollutants show synergistic effects and future research in this field is warranted [60].

Two recent studies provided evidence on how living standards and location may influence the immunological status of children and subsequently their susceptibility to AD. The studies conducted in South Africa, reflect strong differences in living standards between rural and urban areas and revealed significant differences in the immunological status of children living in respective locations [69,70]. The differences in allergy symptoms between these populations were striking (i.e., higher in urban children compared to their rural peers) and the duration of breastfeeding was also of importance (i.e., longer duration in rural communities). The authors provided an immunological basis for this phenomenon. Healthy rural children had the lowest levels of food allergen-specific IgG4. Of interest, independently of AD, the rural children exhibited a generalized type of microinflammation determined by the expression of inflammatory markers [70].

### 4.3. The Dysbiosis Hypothesis

Epidermal barrier dysfunction is invariably present in AD and the skin microbiome is an integral part of the skin barrier [71].

The skin of AD individuals frequently exhibits loss of bacterial diversity and overgrowth of the pathogenic bacteria *S. aureus* [72]. Individuals with AD have greater skin colonization with *S. aureus* than healthy controls (60 to 100% vs. 5–30%, respectively) [73]. In AD patients, the higher skin pH level, reduced levels of FLG and related breakdown products, and lower levels of antimicrobial peptides favor the skin colonization by *S. aureus*, which can promote cutaneous inflammation and AD flare-ups by causing direct proteolytic damage to the epidermal barrier and immune dysregulation [72].

*S. aureus* colonization is associated with AD severity, persistence, and infectious and atopic comorbidities [71,74,75]. *S. aureus* colonization is also associated with greater type 2 inflammation (elevated expression of IL-4, IL-13, IL-17, IL-22, and Thymic Stromal Lymphopoietin, TSLP), allergen sensitization, and tissue damage compared to non-colonized individuals [76,77,78,79]. *S. aureus* activates the immune system by the expression of proteases, toxins, superantigens, and other virulence factors [80,81]. Therefore, *S. aureus* skin colonization is hypothesized to concur with the development of atopic march. Indeed, a recent experimental study showed that the enterotoxin-producing *S. aureus* strain can cause allergen-induced excessive lung inflammation and airway hyperreactivity via an IL-17A-dependent mechanism, in addition to enhancing type 2 inflammatory responses [81].

Tsilochristou et al. [82] recently showed that skin *S. aureus* colonization was significantly associated with AD severity and specific IgE sensitization to egg and peanuts, regardless of the severity of eczema. In addition, *S. aureus* was associated with a more persistent food allergy to egg and peanut [83].

Recent evidence points towards the importance of commensal microbiome composition in early life on the risk of atopic conditions development [84]. In addition to *S. aureus* colonization, the skin of AD patients is often depleted of commensal coagulase-negative staphylococci (CoNS), which could selectively inhibit *S. aureus* through the production of bacteriocins and antimicrobial peptides [85]. A recent small prospective study showed that skin CoNS species were significantly less abundant at 2 months in those infants who developed AD at 1 year compared to those who did not develop AD [86]. A recent human trial has investigated the safety and potential benefits of *S. hominis A9*, a commensal CoNS isolated from the skin of healthy individuals, as a topical bacteriotherapy for AD with promising results [87].

In addition to skin dysbiosis, there is accumulating evidence on the role of gastrointestinal dysbiosis in the development of AD and atopic march, which is extensively reviewed elsewhere [84,88]. The gut microbiota has a major role in shaping and regulating the immune response and consequently the susceptibility to develop immune-mediated disorders, including AD and allergic diseases [84,88].

## 5. Conclusions

It is now acknowledged that atopic march is not a single path, but an umbrella term for multiple trajectories, because atopic diseases are heterogeneous conditions resulting from complex and not fully known interactions between genetic, environmental, and epigenetic factors. Because allergic diseases involve a considerable socio and economic burden all efforts to prevent these diseases are strongly important. AD is often the first manifestation of an atopic march. Improved understanding of the pathogenetic mechanism leading to AD is warranted as this could represent a turning point in the prevention of atopic march. There is a strong need for studies investigating a multifaceted preventive approach targeting both the skin barrier and the environment early in life.

## Data Availability

Not applicable.
